# Deep Learning Based Automatic Grape Downy Mildew Detection

**DOI:** 10.3389/fpls.2022.872107

**Published:** 2022-06-09

**Authors:** Zhao Zhang, Yongliang Qiao, Yangyang Guo, Dongjian He

**Affiliations:** ^1^College of Mechanical and Electronic Engineering, Northwest A&F University, Xianyang, China; ^2^College of Electronic and Electrical Engineering, Baoji University of Arts and Sciences, Baoji, China; ^3^Key Laboratory of Agricultural Internet of Things, Ministry of Agriculture and Rural Affairs, Northwest A&F University, Xianyang, China; ^4^Shaanxi Key Laboratory of Agricultural Information Perception and Intelligent Service, Northwest A&F University, Xianyang, China; ^5^Faculty of Engineering, Australian Centre for Field Robotics (ACFR), The University of Sydney, Sydney, NSW, Australia

**Keywords:** grape downy mildew, disease detection, deep learning, attention mechanism, data augmentation, digital agriculture

## Abstract

Grape downy mildew (GDM) disease is a common plant leaf disease, and it causes serious damage to grape production, reducing yield and fruit quality. Traditional manual disease detection relies on farm experts and is often time-consuming. Computer vision technologies and artificial intelligence could provide automatic disease detection for real-time controlling the spread of disease on the grapevine in precision viticulture. To achieve the best trade-off between GDM detection accuracy and speed under natural environments, a deep learning based approach named YOLOv5-CA is proposed in this study. Here coordinate attention (CA) mechanism is integrated into YOLOv5, which highlights the downy mildew disease-related visual features to enhance the detection performance. A challenging GDM dataset was acquired in a vineyard under a nature scene (consisting of different illuminations, shadows, and backgrounds) to test the proposed approach. Experimental results show that the proposed YOLOv5-CA achieved a detection precision of 85.59%, a recall of 83.70%, and a mAP@0.5 of 89.55%, which is superior to the popular methods, including Faster R-CNN, YOLOv3, and YOLOv5. Furthermore, our proposed approach with inference occurring at 58.82 frames per second, could be deployed for the real-time disease control requirement. In addition, the proposed YOLOv5-CA based approach could effectively capture leaf disease related visual features resulting in higher GDE detection accuracy. Overall, this study provides a favorable deep learning based approach for the rapid and accurate diagnosis of grape leaf diseases in the field of automatic disease detection.

## 1. Introduction

Grape as an important fruit crop makes a large economic income contribution in many countries (Liu et al., 2020; Zhou et al., 2021). As the grape grows in a natural condition, diseases will often appear on the leaves due to the complex weather condition and changing surrounding environments. Grape downy mildew (GDM) is one of the serious diseases caused by the oomycete pathogen Plamopara viticola, which seriously affects the growth of the grapes, causes a decrease in quality and yield, and results in huge economic losses in the grape industry (Chen et al., 2020; Ji et al., 2020). Downy mildew often happened in wet and rainy areas in spring and summer, it is initiated at the stomata on the underside of the leaf, and then on the whole leaf (Chen et al., 2020). Monitoring grape leaf health and detecting pathogen are essential to reduce disease spread and facilitate effective management practices. Grape leaf diseases are currently controlled by repetitive fungicide treatments throughout the season. Reducing the treatment costs is a major challenge from both environmental and economic views. Timely detection and treatment at the initial stage of downy mildew infection (Adeel et al., 2019) is a good solution to control and cut down the spread of downy mildew in a large area. Therefore, if an automatic detection method can be achieved when the spots appear, the leaf disease control plan can be made to control the diseases, guarantee the grape plant health, and improve the quality and yield of the grapes. Vision based detection approaches have been developed to detect plant diseases, which is performed by extracting visual features (e.g., texture, shape, and color of leaf lesions) of leaf images and using models (e.g., support vector machine, linear regression) to recognize and detect the diseases (Tang et al., 2020; Hernández et al., 2021). Zhu et al. (2020) identified grape diseases using image analysis and BP neural networks. Chen et al. (2020) developed and compared several generalized linear models to predict the probability of high incidence and severity in the Bordeaux vineyard region. Abdelghafour et al. (2020) detected downy mildew symptoms using proximal color imaging and achieved 83% pixel-wise precision. However, the traditional image processing technology needs to manually extract the leaf disease characteristics, which is often time-consuming, and easy to miss the best disease prevention time. In addition, under the nature scene (e.g., different illumination, symptoms, camera viewpoints), classical algorithms or models lack robustness and cannot achieve stable detect performance.

Many scholars have proposed approaches for earlier plant disease detection and monitoring of the disease symptoms (Mutka and Bart, 2015). At the earlier stage, the human-crafted features such as texture, color, or shape characteristics are extracted from RGB or hyper-spectral plant leaf images for identifying the plant diseases (Mahlein, 2016). For example, Atanassova et al. (2019) proposed spectral data based classification models to predict the infection in plants, which achieved over 78% accuracy. Waghmare et al. (2016) proposed an automatic grape diseases detection system using the extracted color Local Binary Pattern (LBP) features. Mohammadpoor et al. (2020) proposed a support vector machine for grape fanleaf virus detection and achieved 98.6% average accuracy. However, this kind of method mostly depends on selected features and their extraction is easy to be influenced by the camera viewpoints, shadows, and lighting.

In recent years, deep learning methods such as convolutional neural networks (CNN) have been widely implemented in leaf disease detection, scene perception, and smart agriculture. Variates CNN based detection methods have been proposed for leaf disease recognition and monitoring (Liu et al., 2020). Ferentinos (2018) proposed convolutional neural network architectures to identify healthy or diseased plants. Arsenovic et al. (2019) developed a two-stage architecture of neural networks to classify plant disease and achieved an accuracy of 93.67%. Zhang et al. (2019a) proposed an AlexNet based cucumber disease identification approach, achieving 94.65% recognition accuracy. Ji et al. (2020) proposed CNN based approach to classify common grape leaf diseases and obtained average classification accuracy of 98.57%. Liu et al. (2020) proposed Inception convolutional neural network (DICNN) for identifying grape leaf diseases and realized an overall accuracy of 97.22% on single-leaf datasets. Thet et al. (2020) used an improved VGG16 model that achieved 98.4% classification accuracy for five different leaf diseases. Tang et al. (2020) classified grape disease types using lightweight convolution neural networks and channel-wise attention, which achieved 99.14% accuracy. Liu and Wang (2020) improved the YOLOv3 model to directly generate the bounding box coordinates for tomato diseases and pests detection, which achieved a detection accuracy of 92.39%. According to these studies, CNNs can learn advanced robust features of leaf diseases directly from original images, outperforming the traditional feature extraction approaches. Yu and Son (2020) proposed a leaf spot attention mechanism to increase apple leaf disease discriminative power and enhance the identification performance. Hernández and Lopez (2020) developed Bayesian deep learning techniques and an uncertainty probabilistic programming approach for plant disease detection.

With the continuous development of smart sensors, big data, and cloud computing, many automatic approaches have been proposed to identify and detect plant leaf diseases (Vishnoi et al., 2021). The rapid development of artificial intelligence and the Internet of Things (IoT) has significantly facilitated automatic disease detection (Zhang et al., 2020). Using deep learning models and noninvasive sensors to identify plant diseases has drawn more attention in the field of precision agriculture and plant phenotyping (Nagaraju and Chawla, 2020; Singh et al., 2020). Hernández et al. (2021) investigated hyperspectral sensing technologies and artificial intelligence applications for assessing downy mildew in grapevine under laboratory conditions. Gutiérrez et al. (2021) differentiated downy mildew and spider mite in grapevine under field conditions using the CNN model. Liu et al. (2021) proposed Hierarchical Multi-Scale Attention Semantic Segmentation (HMASS) to identify GDM infected regions, and the calculated infection severity percentage was highly correlated (*R* = 0.96) with the human field assessment.

Choi and Hsiao (2021) classified Cassava leaf diseases using the Residual Network. Zhang et al. (2021) developed a multi-feature fusion Faster R-CNN model and achieved 83.34% detection accuracy for soybean leaf disease. Dinata et al. (2021) proposed CNN based approach for 6 types of strawberry disease classification and achieved 63.7% accuracy. Abbas et al. (2021) detected tomato plant disease using transfer learning and C-GAN synthetic images, which achieved 99.51% accuracy. Cristin et al. (2020) proposed a deep neural network based Rider-Cuckoo Search Algorithm and achieved 87.7% plant disease detection accuracy. Roy and Bhaduri (2021) proposed deep learning-based multi-class plant disease and achieved 91.2% mean average precision. However, most of these methods are only tested in experimental situations, which need to be verified on the complex background situation.

Despite deep learning based approaches demonstrating its facilitate in GDM detection, the detection accuracy and speed restricted its application in autonomous viticulture management. Plant leaf disease detection in the real vineyard is facing many challenges, such as the small difference between the lesion area and the background, different scales of the spots, variation of symptoms, and camera viewpoints (Liu and Wang, 2021). Also, light changing in a real complex natural environment further increased the difficulty to achieve high detection accuracy. Therefore, real-time and accurate detection of grape downy mildew is of great significance for the scientific management and control of grape diseases in precision vineyard farming.

Recently, the attention mechanisms such as Squeeze-and-Excitation Networks (SE) (Hu et al., 2018), Convolutional block attention module (CBAM) (Woo et al., 2018), and CA (Hou et al., 2021) have been widely used to enhance the deep learning model performances. SE simply squeezes each 2D feature map to efficiently build interdependencies among channels (Hu et al., 2018). CBAM introduces spatial information encoding via convolutions with large-size kernels and gathers channel-wise and spatial-wise attention sequentially. The recently proposed CA adopts different spatial attention mechanisms and designs advanced attention blocks. Zhang et al. (2019b) applied an attention mechanism to object detection networks, enhancing the impact of significant features and weakening background interference. Experimental results show that the proposed approach achieved an object detection accuracy of 75.9% on PASCAL VOC 2007, which is 6% higher than Faster R-CNN. Liu et al. (2019) presented a deep neural network architecture based on information transmission and attention mechanisms. Zhao et al. (2021) diagnosed tomato leaf disease using an attention module improved network, which achieved 96.81% average identification accuracy on the tomato leaf diseases dataset. Ravi et al. (2021) integrated the attention module into the EfficientNet model to locate and identify the tiny infected regions in the Cassava leaf. The proposed method achieved better performance than non-attention-based CNN pre-trained models. Wang et al. (2021b) proposed a Fine-Grained grape leaf disease recognition method using a lightweight attention network, which can efficiently diagnose orchard grape leaf diseases with low computing cost. The above studies have demonstrated that attention mechanisms could enhance feature extraction ability for leaf disease detection and identification.

In this study, to improve GDM detection accuracy in the natural grape farm environment, we proposed YOLOv5-CA based GDM detection approach by combing YOLOv5 and CA mechanism. Different scales of image features were extracted through CNN layers of YOLOv5, and these features were weighted by CA for GDM detection. By using CA, the features' effectiveness for GDM detection is highlighted and those less effective features are suppressed. The proposed YOLOv5-CA based GDM detection is tested on our acquired grape leaf image dataset.

The remaining part of the article is organized as follows. Section 2 illustrates the used datasets, the proposed approaches, and evaluation indicators. Experimental results are presented in Section 3. Discussions of the performance are presented in Section 4. Finally, conclusions and future areas for research are given in Section 5.

## 2. Material and Methodology

### 2.1. Plant Material and Image Acquisition

**Figure 1:**
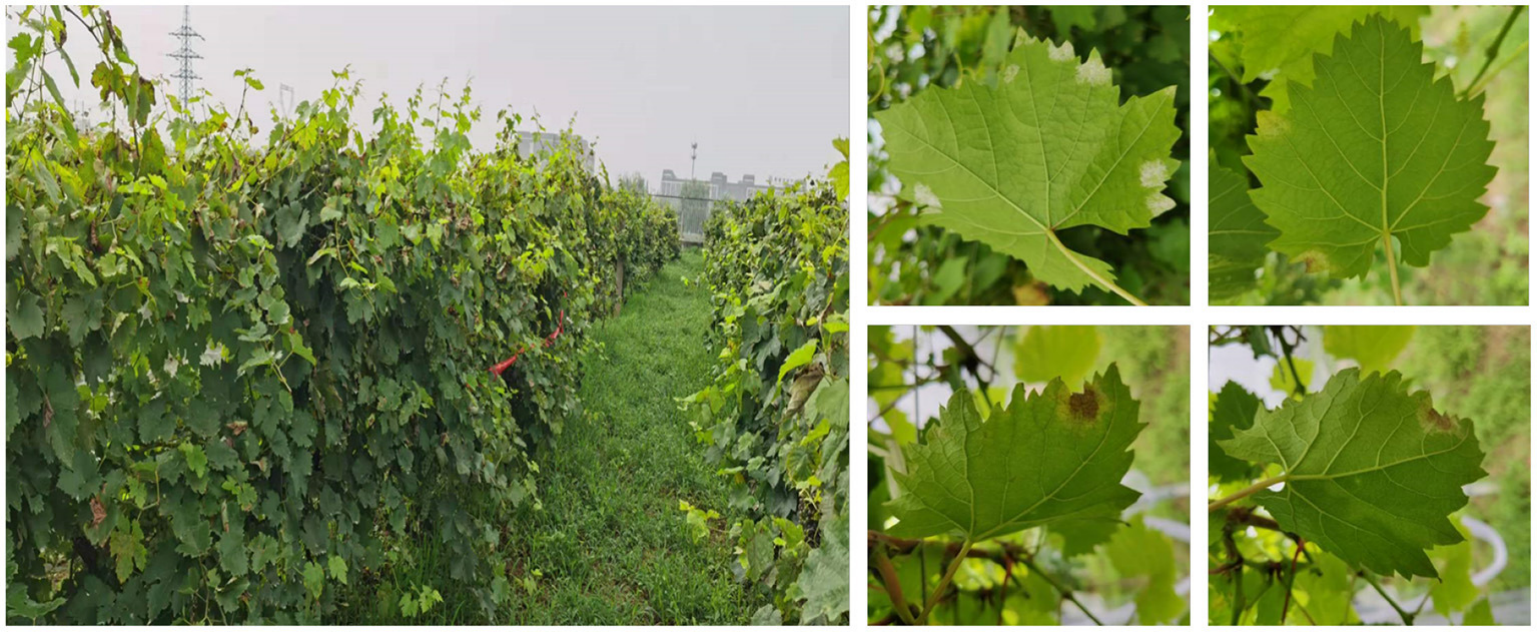
Commercial vineyard and acquired images under natural light conditions.

Grape leaf image data were acquired in a commercial vineyard located in the college of Enology, Northwest A&F University, north of China (Yangling, Shaanxi Province). The vineyard manifested downy mildew (Plasmopara viticola) in many plants. Images were taken manually for several days (each day is from 8:00 a.m. to 16:00 p.m.) in early August on a partly cloudy day (Figure 1). The used camera is a Canon EDS 1200D (a field of view of approximately 504 mm horizontally and 360 mm vertically), and the external conditions for shooting are automatic mode. There is approximately 30 cm between the camera lens and the grape leaves.

A total of 820 leaf samples were collected from different lights, leaf overlapping, and disease severity. The dataset is challenging considering the complex background, occlusions, different disease spot-areas, and shadow influence. Figure 1 shows images of diseased leaves in a typical complex environment in the dataset. Downy mildew first appears as brown patches. These patches gradually spread and a leaf that is severely affected may have a reduced yield with a shorter lifetime and fruits with a small size.

To validate the proposed YOLOv5-CA based GDM detection approach, the randomly selected 500 leaf images were used as training datasets, while the remaining 320 images were used as testing data. For experiment testing, the LabelImg annotation tool (Tzutalin, 2015) was used to manually label the leaf disease areas.

### 2.2. YOLOv5-CA Based GDM Detection

**Figure 2:**
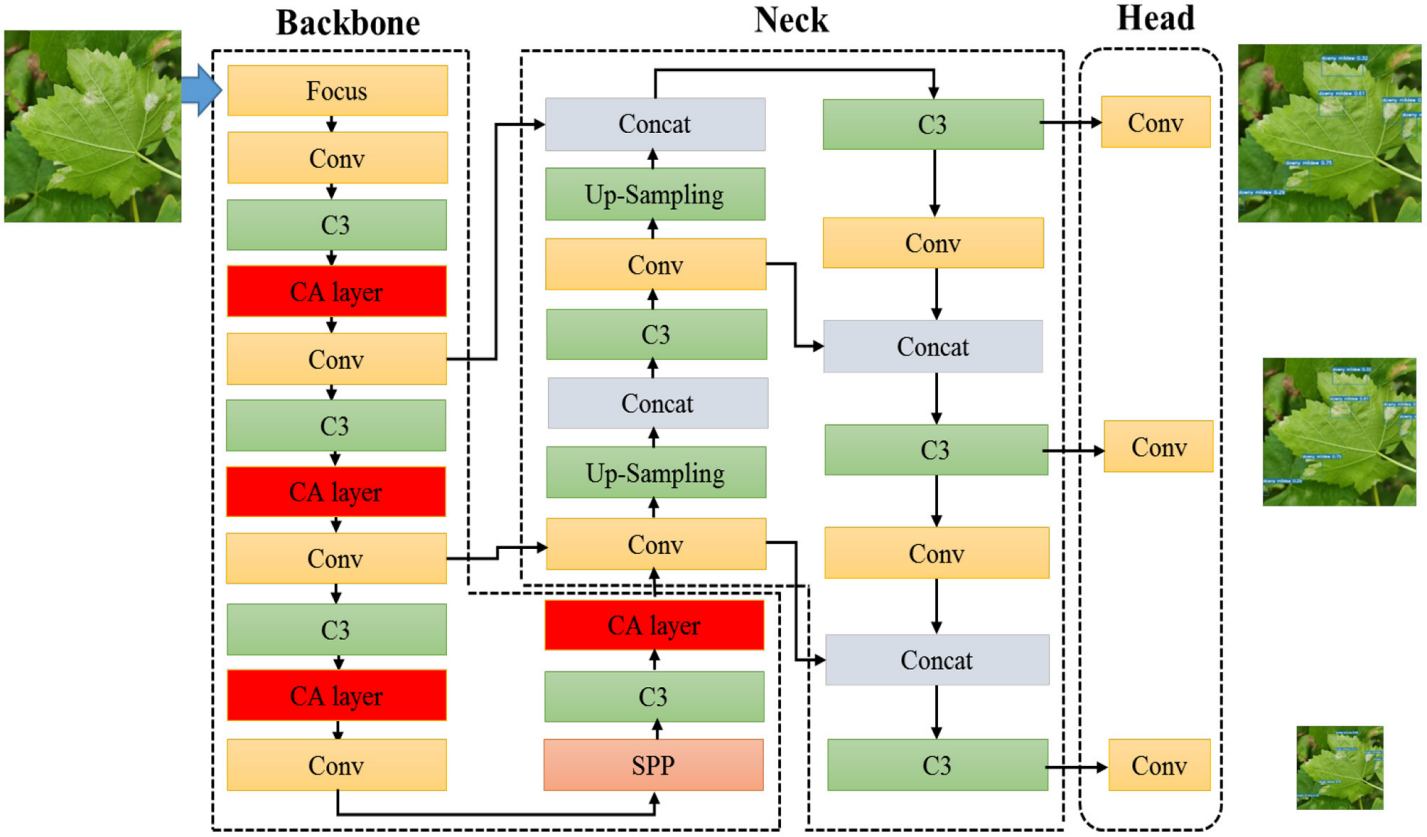
The architecture of the proposed YOLOv5-CA based GDM detection.

In order to make YOLOv5 more suitable for GDM detection in complex natural scenarios such as complex background, occlusions, different disease spot-areas, and shadow influence, YOLOv5-CA based GDM detection approach is proposed to improve the GDM detection performance for real farming applications. Grape leaves' RGB images were acquired under field conditions from a commercial vineyard. These collected images contain healthy and downy mildew infected leaves. Then detection model YOLOv5-CA was trained to identify the GDM infected regions. As shown in Figure 2, the proposed YOLOv5-CA approach extracted features using YOLOv5 and learned key features through CA, enhancing the feature extraction ability and improving the leaf disease detection performance. As YOLOv5 could adjust the width and depth of the backbone network according to application requirements, for GDM detection, moderate model parameters (i.e., width and depth parameters are 0.75 and 0.67, respectively) were used to achieve reasonable detection speed.

YOLOv5-CA network is mainly composed of backbone part, neck network, and head part: 1) The backbone of YOLOv5 is responsible for extracting image features, which includes several different layers types such as Focus, Conv, C3, CA, and Spatial Pyramid Pooling (SPP) layer. 2) The neck module generates a feature pyramid based on the PANet (Path Aggregation Network) (Liu et al., 2018). It is a series of feature aggregation layers of mixed and combined image features, enhancing the ability to detect objects with different scales by fusing low-level spatial features and high-level semantic features. 3) The head module generates detection boxes, indicating the category, coordinates, and confidence by applying anchor boxes to multi-scale feature maps from the neck module. The proposed YOLOv5-CA boosts the detection ability of different GDM infection regions through an attention mechanism, which provides a feasible GDM detection and monitoring solution for automatic disease control.

#### 2.2.1. Backbone of YOLOv5-CA

The backbone of the YOLOV5-CA object detector mainly contains Focus, Conv, C3, CA, and Spatial Pyramid Pooling (SPP) layer. The features from deeper layers are more abstract and semantic, while the low-layer features contain spatial information and fine-grained features. For an input image, the Focus module rearranged it through stridden slice operations in both width and height dimensions, which reduces model calculation time. C3 module contains three convolutions and is used to extract the deep features of the image. The following SPP is used to improve the receptive field of the network by converting any size of the feature map into a fixed-size feature vector. SPP (He et al., 2015) concatenates layer outputs with different kernel sizes (e.g., 13 × 13, 9 × 9, 5 × 5) to boost multi-scale image feature representation ability. All the convolutions utilize Swish activation:


(1) $$\begin{array}{l} Swish\left(x\right)=x\times \sigma \left(x\right) \end{array}$$

where σ denotes the sigmoid function.

In our study, we integrated the CA layer into the YOLOv5 backbone, CA layer factorizes channel attention into two 1D feature encoding processes and preserves the precise positional information, which augments the representations of the leaf disease regions.

#### 2.2.2. Neck of YOLOv5-CA

The neck structure used in YOLOv5-CA is a PANet (Liu et al., 2018), which fuses the information of all layers to aggregate features by combing bottom-up pyramid and element-wise max operations. PANet combines convolution features of different layers for images, thus the useful information in each feature layer can be directly propagated to the following subnetwork. By this, PANet can not only realize the abstract description of large objects but also retains the feature details of small objects. In addition, C3 modules are also added at this stage to enhance the feature fusion capability. Through the neck part, the features of infected areas can be extracted to maintain the detection performance.

#### 2.2.3. CA Layer

In terms of GDM detection, because the GDM is randomly distributed in the grape leaf, there is inevitably a mix of overlapping occlusion, and the GDM infection regions account for a relatively small percentage of the images, resulting in missed and mis-detected. In our study, a plug-and-play CA layer was introduced to assist YOLOv5 focused on key disease-related features, and improve the detection accuracy.

The CA layer embeds the location-aware information into the channel attention simultaneously, which increases the spatial range of attention and avoids a lot of computational overhead (Hou et al., 2021). CA layer can be regarded as a computational unit that enhances the representation ability of the learned features. For any intermediate feature $X=\left[x1,x2,\cdots x_{c}\right]\in {\mathbb{R}}^{C\times H\times W}$, CA could outputs a transformed feature with augmented representations *Y* = [*y*1, *y*2, ⋯ , *y*_*c*_] of the same size to *X*.

**Figure 3:**
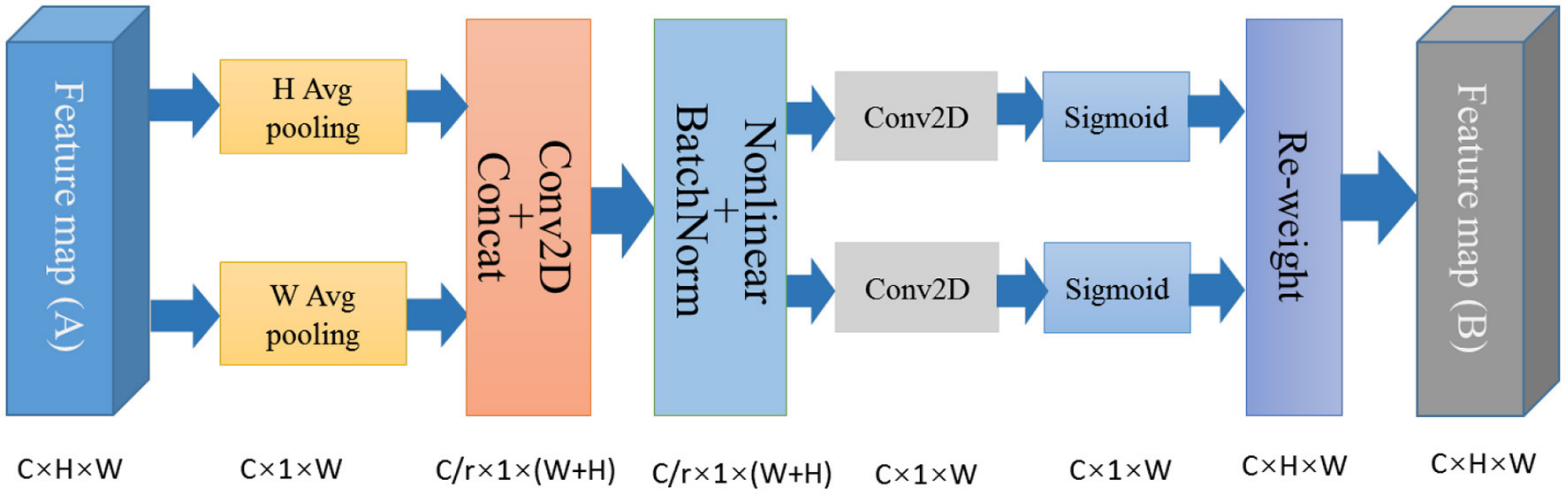
Schematic of coordinate attention module.

As shown in Figure 3, the CA mechanism can be divided into two parts: the coordinate information embedding part (encodes the information of the channels in the horizontal and vertical coordinates) and the coordinate attention generation part (captures the positional information and generates the weight values).

#### 2.2.4. Coordinate Information Embedding

Attention mechanisms have been demonstrated helpful to enhance the overall performance of deep learning models (Chorowski et al., 2015). The attention mechanism can be regarded as a feature weighting scheme, which helps the deep learning model to pay more attention to the task-related information, and suppress or ignore the less-contribution features (Li et al., 2020; Mi et al., 2020). Through this, the attention mechanism strengthens the deep learning model's learning ability and boosts performance (Niu et al., 2021). In recent years, attention mechanisms based on deep learning networks have been applied to a wide variety of computer vision tasks such as image classification, object detection, and image segmentation (Qiao et al., 2019, 2021). Wang et al. (2021a) developed a deep attention module for vegetable and fruit leaf plant disease detection. Kerkech et al. (2020) used a fully convolutional neural network approach to classify Unmanned Aerial Vehicle (UAV) image pixels for detecting mildew disease.

It is known that channel attention could increase the value of the important channel while punishing the non-significant channels, however, channel attention is difficult to preserve positional information (Zhang et al., 2018). To capture precise positional information, the global average pooling was factorized into the average pooling from two directions of each channel. Specifically, given the input *X*, two spatial extents of pooling kernels (H, 1) and (W, 1) were used to encode each channel along the horizontal coordinate and the vertical dimensions, respectively. The output of the *c*-*th* channel along height *h* and width *w* dimensions can be formulated as:


(2) $$\begin{array}{l} \begin{array}{c} z_{c}^{h}\left(h\right)=\frac{1}{W}\underset{0\leq i<W}{\sum }x_{c}\left(h,i\right),\\ z_{c}^{w}\left(w\right)=\frac{1}{H}\underset{0\leq j<H}{\sum }x_{c}\left(w,j\right). \end{array} \end{array}$$

where *z*^*h*^ and *z*^*w*^ are the outputs of the transform at *h* direction width *w*, respectively; *x*_*c*_ is the feature map at *c*-*th* channel; *W* and *H* are the width and height dimensions of the feature map separately.

The Equation (2) encodes each channel along with the horizontal and vertical coordinates, preserving the positional information of each channel of feature maps, which facilitates the network to locate the GDM-related visual features precisely.

#### 2.2.5. Coordinate Attention Generation

To further exploit resulting expressive representations, a simple and effective coordinate attention generation was used as the second transformation. Here, the obtained feature maps from the coordinate information embedding stage were concatenated and then sent to a shared 1 × 1 convolution layer. The relevant process is defined as:


(3) $$\begin{array}{l} f=Relu\left(F\left(\left[z^{h},z^{w}\right]\right)\right) \end{array}$$

where [, ] indicates concatenate operation, *F* is 1 × 1 convolution operation; $f\in {\mathbb{R}}^{\frac{C}{r}\times \left(W+H\right)}$ is the output feature map of the ReLU layer, *r* is reduction rate.

Next, the feature map *f* was decomposed into two separate tensors: $f^{h}\in {\mathbb{R}}^{\frac{C}{r}\times H}$ and $f^{w}\in {\mathbb{R}}^{\frac{C}{r}\times W}$. Then the following two 1 × 1 convolution layers for *f*^*h*^ and *f*^*w*^, respectively, are recovered to the same shape as *z*^*h*^ and *z*^*w*^. The operation is formulated as:


(4) $$\begin{array}{l} \begin{array}{c} g^{h}=\sigma \left(F_{h}\left(f^{h}\right)\right),\\ g^{w}=\sigma \left(F_{w}\left(f^{w}\right)\right). \end{array} \end{array}$$

where σ is the sigmoid activation function, and *F*_*h*_ and *F*_*w*_ are the convolution manipulation for *f*^*h*^ and *f*^ω^ separately.

The obtained feature maps *g*^*h*^ and *g*^*w*^ are then expanded and used as attention weights for the horizontal and vertical coordinates, respectively. This operation can enhance the effective leaf disease related features and reduce the influence of less important information. The reweighing process of the original input feature map can be defined as:


(5) $$\begin{array}{l} y_{c}\left(i,j\right)=x_{c}\left(i,j\right)\times g_{c}^{h}\left(i\right)\times g_{c}^{w}\left(j\right). \end{array}$$

where *y*_*c*_ is the *c*-*th* channel in the generated feature map *y* of the attention block.

### 2.3. YOLOv5-CA Model Training for GDM Detection

#### 2.3.1. Network Training Parameters

In our study, the experimental platform is based on a computer equipped with an NVIDIA RTX 1080Ti GPU, Ryzen 7 3600 CPU@3.6 GHz. The proposed GDM detection approach was implemented using Pytorch.

In addition, to verify the effectiveness of the YOLOv5-CA based GDM detection approach, Faster R-CNN (Ren et al., 2015), YOLOv4 (Bochkovskiy et al., 2020), and YOLOv5 (Tzutalin, 2015) were also used for comparison. Faster R-CNN generates regions of interest (RoIs) candidates and then classifies them into objects (and background) and refines the boundaries of those regions. YOLOv4 and YOLOv5 are the two widely used detection methods from the YOLO series (Redmon et al., 2016).

For network training, the network's input size was set to 416 × 416 × 3, the training epoch was set to 1000, batch size was set to 16, and the learning rate was 0.0013. The momentum factor (momentum) was set to 0.937, the initial learning rate was 1 × 10^−5^ and the decay rate of weight was set to 0.001. The other parameters of each network are their default settings. In the training process, the network predicts the bounding box based on the initial anchor box. The gap between the prediction and ground truth was calculated to update the network in reverse and adjusts the network parameters. After training, the weight file of the detection model obtained was saved.

#### 2.3.2. Network Loss Function

YOLOV5-CA automatically updates the best bounding box for GDM detection during the training process. The default optimization method of the model is the gradient descent method. The loss function *L*_*loss*_ used in YOLOv5-CA includes bounding box location loss *L*_*Ciou*_, confidence loss *L*_*conf*_ and classification loss *L*_*cls*_:


(6) $$\begin{array}{l} L_{loss}=L_{cls}+L_{conf}+L_{CIoU} \end{array}$$

Classification loss *L*_*cls*_ computes the loss of class probability using Cross Entropy:


(7) $$L_{cls}=\sum_{i=0}^{s^{2}}\ell_{i,j}^{obj}\sum_{c\in classes}\left[\overset{\wedge }{p_{i}}(c)log(p_{i}\left(c\right))+(1-\overset{\wedge }{p_{i}}(c))log(1-p_{i}(c))\right]$$

where $\ell_{i}^{obj}$ is used to judge whether there is an object center. ${\hat{p}}_{i}\left(c\right)$ is the probability of class *c*; *p*_*i*_(*c*) is the probability of predicted box that belongs to class *c*.

Confidence loss *L*_*conf*_ penalizes object confidence error if that predictor is responsible for the ground truth box, which is computed using mean squared error:


(8) $$\begin{array}{l} L_{conf}=\sum_{i=0}^{s^{2}}\sum_{j=0}^{B}\ell_{i,j}^{obj}\left[\overset{\wedge }{C_{i}}log(C_{i})+(1-\overset{\wedge }{C_{i}})log(1-C_{i})\right]\\ +{\text{λ}}_{noobj}\sum_{i=0}^{s^{2}}\sum_{j=0}^{B}\ell_{i}^{noobj}\left[\overset{\wedge }{C_{i}}log(C_{i})+(1-C_{i})log(1-\overset{\wedge }{C_{i}})\right] \end{array}$$

where λ_*noobj*_ represents the weight of the classification error, *S* is the number of grids, and *B* is the number of prior boxes in each grid; *C*_*i*_ is the confidence of the predicted box; Ĉ_*i*_ is the confidence of the ground-truth (Ĉ_*i*_ is always 1).

The *L*_*Ciou*_ computes the loss related to the predicted bounding box and ground truth, it can be defined as follows:


(9) $$\{\begin{array}{l} L_{CIoU}=1-IoU+\frac{\rho^{2}\left(b,b^{gt}\right)}{e^{2}}+\frac{\nu^{2}}{(1-IoU)+\nu }\\ IoU=\frac{\left|b\cap b^{gt}\right|}{\left|b\cup b^{gt}\right|} \end{array}$$

where *v* represents the coincidence degree of the two frame aspect ratios, *b* and *b*^*gt*^ are the center coordinates of the prediction box and the real box respectively; ρ is the Euclidean distance between the two center points, and *e* represents the diagonal distance of the smallest closed area containing both the prediction and real boxes. *IoU* means the ratio of the intersection and union of the prediction bounding box and the actual bounding box.

#### 2.3.3. Performance Evaluation

The used performance evaluation indicators for GDM detection include precision, recall, *F*_1_-score, mAP (mean average precision), and FPS (frame per second). Precision shows the ability of the model to accurately identify targets; recall reflects the ability of the model to detect targets; the *F*_1_-score is a harmonic mean of the precision and recall; FPS is the average inference speed. The *F*_1_-score is the reconciled mean of precision and recall, taking into account both the precision and recall of the classification model. Based on tp (the number of hlcorrectly detected downy mildew areas), fp (the number of incorrectly detected downy mildew areas), and fn (the number of disease regions that are incorrectly identified as background), the relevant calculation equations are as follows:


(10) $$\begin{array}{l} Precision=\frac{tp}{tp+fp}\times 100\% \end{array}$$

(11) $$\begin{array}{l} Recall=\frac{tp}{tp+fn}\times 100\% \end{array}$$

(12) $$\begin{array}{l} F_{1}=2\times \frac{Precision\times Recall}{Precision+Recall}\times 100\% \end{array}$$

From the values of precision and recall, a precision-recall curve can be plotted to observe their distribution. The value of AP is the area under the precision-recall curve, and a larger value means better model performance. mAP@0.5 is the average value of precision under different recall values when the intersection over union (IoU) is 0.5. The calculation of mAP is as follows:


(13) $$\begin{array}{l} mAP=\frac{1}{n}\overset{N}{\underset{k=1}{\sum }}AP_{k} \end{array}$$

where *N* denotes the number of disease types (*N* is 1 in our study).

## 3. Experimental Results

### 3.1. Comparison of Different Object Detection Algorithms

There are varieties of deep learning based detection methods, in order to verify the effectiveness of the proposed method for GDM detection, three popular detection algorithms—Faster R-CNN, YOLOv4, and YOLOv5 were compared. The GDM detection results were presented in Table 1.

**Table 1:** Comparison of different GDM methods.

**Method**	**Precision (%)**	**Recall (%)**	***F*_1_ (%)**	**mAP@0.5 (%)**	**FPS (Frame/s)**
Faster R-CNN	79.97	87.80	83.70	80.65	35.90
YOLOv4	82.69	83.63	83.15	82.65	75.20
YOLOv5	85.35	81.45	83.36	87.41	84.74
YOLOv5-CA	85.59	83.70	84.63	89.55	58.82

In Table 1, the proposed YOLOv5-CA based approach achieved 85.59% precision, 83.70% recall and 84.63% *F*_1_, and 89.55% mAP, respectively. Compared with the other methods, the proposed YOLOv5-CA GDM detection method is better than that of Faster R-CNN (80.65% mAP), YOLOv4 (82.65 % mAP), and YOLOv5 (87.41% mAP). From these results, it is clear that the CA mechanism of YOLOv5-CA improves the feature representation ability, enhancing the final detection accuracy for identifying the leaf disease areas. Meanwhile, the proposed approach could detect the GDM with a speed of 58.82 frames per second. These results illustrated that the proposed method could achieve high precision with a fast speed to meet real-time requirements, which is favorable for the deployment of the GDM detection model in spraying robots for the plant diseases control in smart vineyard farming.

### 3.2. Qualitative GDM Detection Comparison

**Figure 4:**
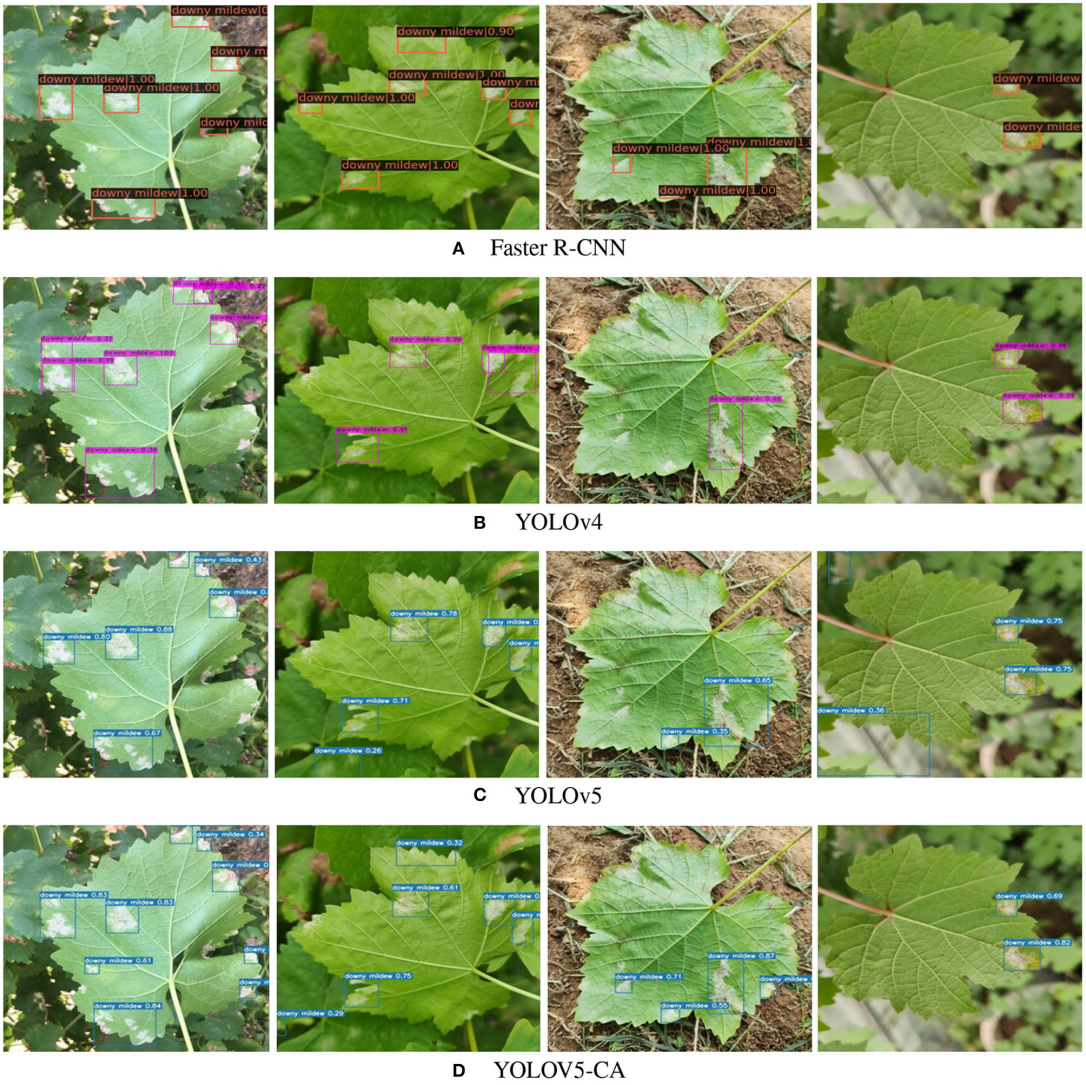
Examples of different GDM detection attention methods. **(A)** Faster R-CNN, **(B)** YOLOv4, **(C)** YOLOv5, and **(D)** YOLOV5-CA.

Figure 4 demonstrates the comparison of different methods' qualitative results on our acquired grape leaf dataset. It can be seen that the proposed YOLOv5-CA could detect GDM at different leaf parts (e.g., leaf edge, the leaf central parts). Especially, the proposed YOLOv5-CA method could detect less obvious GDM lesions on the leaves, which outperformed the other methods such as Faster R-CNN, YOLOv4, and YOLOv5. The main reason could be that the CA mechanism strengthens the feature representation ability, which enhances the GDM detection performance.

**Figure 5:**
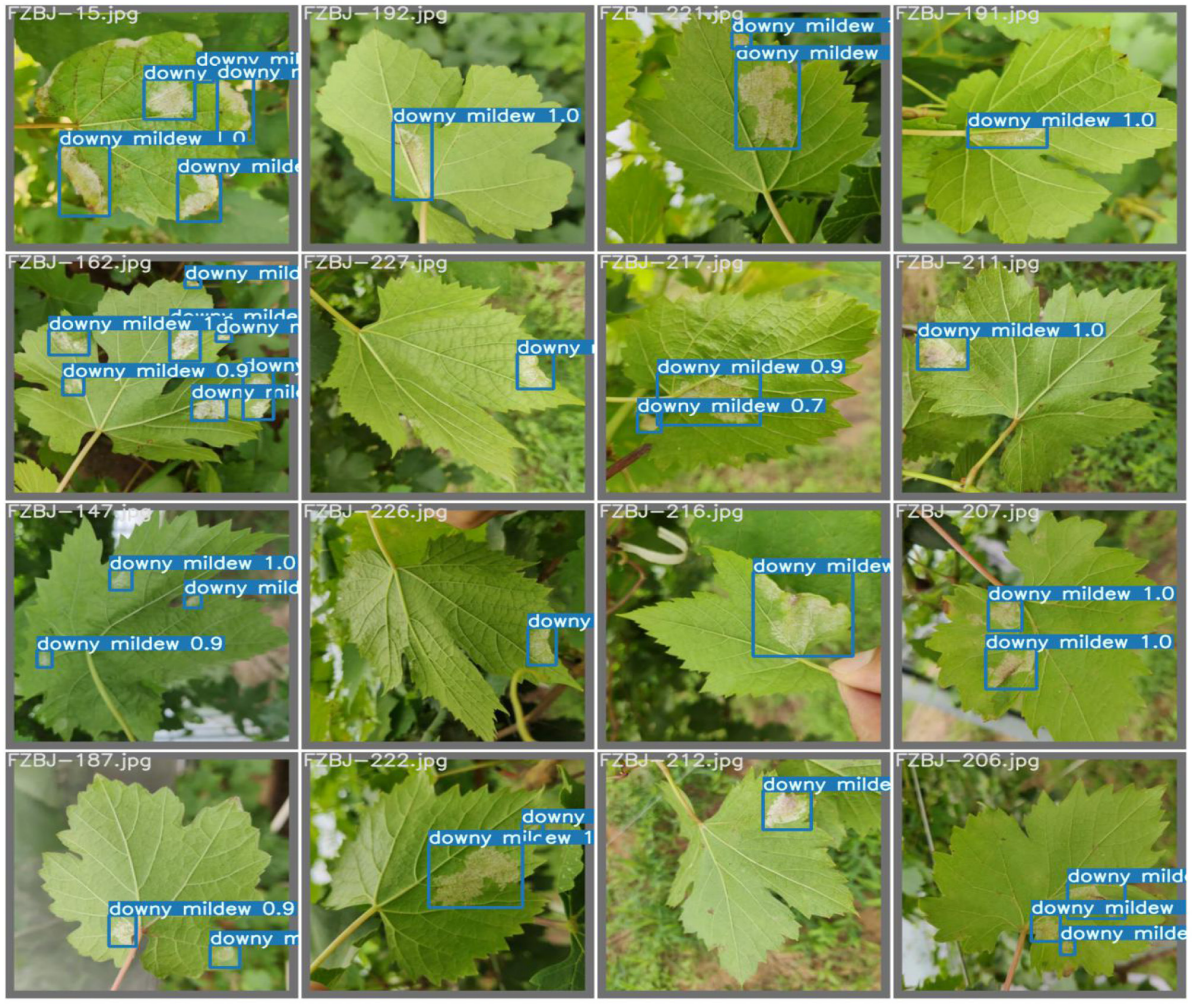
Examples of YOLOv5-CA based GDM detection results.

Additionally, more examples of YOLOv5-CA based GDM detection are presented in Figure 5. It can be seen that the GDM infected regions are well detected (blue bounding box) under complex background, especially, YOLOv5-CA could well detect the GDM regions nearby the leaf edge and petioles. It also can be noted that the YOLOv5-CA could detect both large and small GDM regions. The main reason is that the YOLOv5-CA makes the network pay more attention to the GDM-related visual features, reducing the false or mis-detection cases. The good detection performance of YOLOv5-CA provides valuable information for automatic disease control.

### 3.3. Influence of Different Network Input-Sizes on GDM Detection

The network input size is one factor that would influence the GDM detection performance. Here, we also investigate different input-sizes' influence on YOLOv5-CA based GDM detection. In Table 2, five typical network input sizes, namely, 112 ×112, 224 ×224, 320 ×320, 416 ×416, and 512 ×512 were compared in terms of GDM detection performance.

**Table 2:** Grape downy mildew Detection performance with different network input sizes.

**Network input size**	**Precision (%)**	**Recall (%)**	***F*_1_ (%)**	**mAP@0.5**	**FPS (Frame/s)**
112 ×112	80.32	72.76	76.35	76.71	102.04
224 ×224	83.73	79.32	81.47	82.63	92.63
320 ×320	84.75	84.32	84.53	85.25	76.92
416 ×416	85.59	83.70	84.63	89.55	58.82
512 ×512	86.71	82.80	84.71	87.89	45.45

According to Table 2, the network input with 416 ×416 size achieved 85.59% precision, 83.70% recall, 84.63% *F*_1_-score, and 89.55% mAP@0.5, which outperformed the performance of input size with 112 ×112, 224 ×224, and 512 ×512. This means the proposed YOLOv5-CA could extract and learn the more useful information from the large input size. However, when the network input-size increases to 512 ×512, there is not much performance improvement but significantly increased the processing time and calculating memory size, which is not favorable for fast detection and real applications. By balancing the speed and accuracy, the input size of 416 ×416 was selected in our work for real-time GDM detection.

### 3.4. Data Augmentation for YOLOv5-CA Detection

**Figure 6:**
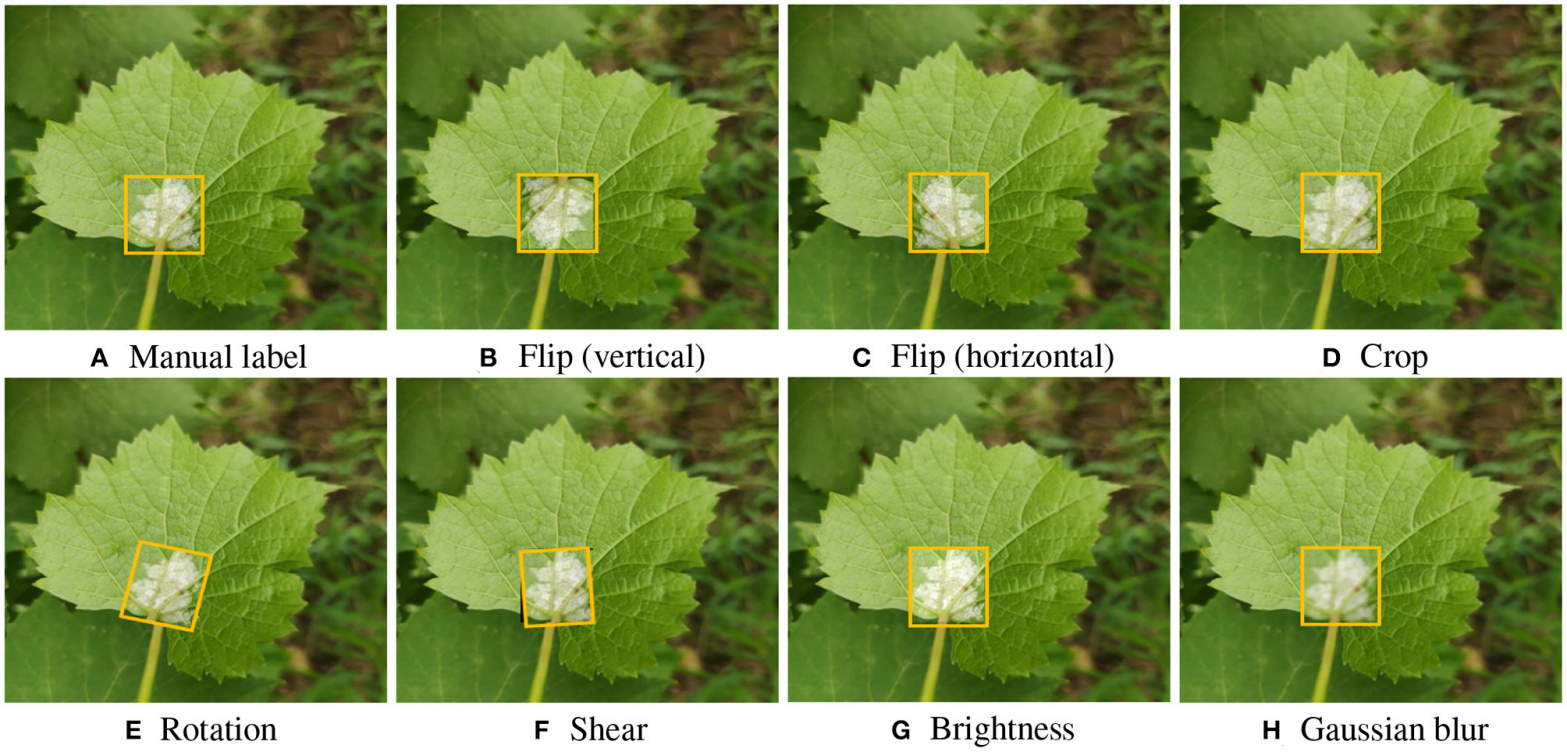
Examples of Bounding box based data augmentation. **(A)** Manual label, **(B)** Flip (vertical), **(C)** Flip (horizontal), **(D)** Crop, **(E)** Rotation, **(F)** Shear, **(G)** Brightness, and **(H)** Gaussian blur.

Offline data augmentation could increase the dataset diversity, explore the network hyperparameters, and finally enhance the accuracy and robustness of the trained model (Zoph et al., 2020; Su et al., 2021). To further improve the GDM detection performance, in our study, bounding box based data augmentation was used. The augmentation technique was only applied to disease areas within the manually labeled bounding boxes. The transformations for data augmentation implemented include: flipping horizontally and vertically, randomly cropping between 0 and 20% of the bounding box, random rotation, random shear of between −15° to +15° horizontally and vertically, random brightness adjustment (between 0 and +10%), and Gaussian blur (between 0 and 5 pixels). The original 500 training images were expanded to 2000 images, and then they were used to train the YOLOv5-CA network, which forces neural nets to optimize hyperparameters and generate a high-robust model. Some augmented bounding boxes on grape leaves can be seen in Figure 6.

As illustrated in Table 3, the data augmentation based YOLOv5-CA detection achieved a precision of 88.82%, a recall of 83.63%, and an *F*_1_-score of 86.15%, which is slightly higher than those without data augmentation. The data augmentation positively influences the model's performance by increasing the size of the dataset and mitigating the over-fitting. The overall improvements demonstrated that the data augmentation module is helpful in the GDM detection, enlarging model learning ability and significantly improving detection performance.

**Table 3:** Comparison of different GDM methods.

**Method**	**Precision (%)**	**Recall (%)**	***F*_1_ (%)**	**mAP@0.5 (%)**
YOLOv5-CA	85.59	83.70	84.63	89.55
YOLOv5-CA (with data augmentation)	88.82	83.63	86.15	90.02

## 4. Discussions

This study presents a deep learning-based pipeline for automatic GDM detection in the vineyard. The grape leaf images acquired directly from the plants under field conditions were used to verify our proposed YOLOv5-CA approach. According to our experimental results presented in Table 1, a precision of 85.59%, a recall of 83.70%, an *F*_1_-score of 84.63%, and a mAP@0.5 of 89.55% with the inference speed of 58.82 frames per second (FPS) was obtained for GDM detection. The detection accuracy of the proposed YOLOv5-CA is superior to that of state-of-the-art methods such as Faster R-CNN, YOLOv4, and YOLOv5. This high accuracy demonstrates the effectiveness of YOLOv5-CA for GDM detection of grapevine leaf images taken under field conditions. There yield results show that it is feasible to model visual symptoms for automatic GDM detection using a combination of the YOLOv5 and the CA mechanism. The proposed YOLOv5-CA automatically finds complex features capable of differentiating leaves with downy mildew symptoms and without any, which provides a precise and effective method for automatic disease detection.

On the other hand, the results presented in Table 2 reveal the appropriate network input size in our experiments is 416 ×416. Additionally, Table 3 compared the GDM detection performance with and without data augmentation, it shows that data augmentation enhances the GDM detection performance. The possible reason is that data augmentation increases the size of the dataset and brings more diversity to leverage the model training.

Although this study mainly focuses on GDM detection, it is suitable for multi-diseases detection (e.g., black spot, powdery mildew) after the model was re-trained with the dataset containing these diseases. As our approach uses RGB images, it would be a restriction for detecting GDM in the very earlier stage (i.e., non-visible symptoms) detection. However, if the multi-spectral images were acquired and used, our proposed YOLOv5-CA could be a potential tool to distinguish downy mildew from other leaf diseases/damage.

## 5. Conclusions and Future Study

To achieve an accurate and real-time intelligent detection of GDM under natural environments, an automatic YOLOv5-CA based detection method was proposed in this study. By combing YOLOv5 and coordinate attention, the GDM related visual features are well focused on and extracted, which boosts the GDM detection performance. Our proposed YOLOv5-CA achieved 85.59% detection precision, 89.55% mAP@0.5 with 58.82 FPS, which outperformed Faster R-CNN, YOLOv4, and YOLOv5. Moreover, the test results showed that the different disease levels of GDM and the illumination influence would not have a great impact on the GDM detection results, indicating the proposed method is feasible for the rapid and accurate detection of GDM. Ablation studies show that a network input size of 416 ×416 is favorable for fast GDM detection, and bounding box-based data augmentation boosts the GDM detection precision by 3.23%. The results exposed in this work indicate that downy mildew in grapevine can be automatically evaluated using artificial intelligence technology.

Overall, our approach achieved a good trade-off between speed and accuracy for GDM, and can be adapted to applications with autonomous-based smart farming. For future study, the multi-spectral information and edge computing will be exploited to further improve detection performance and computational efficiency.

## Data Availability Statement

The original contributions presented in the study are included in the article/supplementary material, further inquiries can be directed to the corresponding authors.

## Author Contributions

ZZ: investigation, methodology, writing-review, and editing. YQ: data curation, methodology, formal analysis, and writing-original draft. YG: writing-review and editing. DH: resources and article revising. All authors contributed to the article and approved the submitted version.

## Funding

This research was funded by the National Key Research and Development Program of China (2019YFD1002500), Ningxia Hui Autonomous Region Key Research and Development Program (2021BEF02015), and Ningxia Hui Autonomous Region Flexible Introduction Team Project (2020RXTDLX08).

## Conflict of Interest

The authors declare that the research was conducted in the absence of any commercial or financial relationships that could be construed as a potential conflict of interest.

## Publisher's Note

All claims expressed in this article are solely those of the authors and do not necessarily represent those of their affiliated organizations, or those of the publisher, the editors and the reviewers. Any product that may be evaluated in this article, or claim that may be made by its manufacturer, is not guaranteed or endorsed by the publisher.
